# A high-throughput system for high-quality tomographic reconstruction of large datasets at Diamond Light Source

**DOI:** 10.1098/rsta.2014.0398

**Published:** 2015-06-13

**Authors:** Robert C. Atwood, Andrew J. Bodey, Stephen W. T. Price, Mark Basham, Michael Drakopoulos

**Affiliations:** Diamond Light Source Ltd, Harwell Science and Innovation Campus, Didcot OX11 0QX, UK

**Keywords:** tomography, computation, X-ray, data processing

## Abstract

Tomographic datasets collected at synchrotrons are becoming very large and complex, and, therefore, need to be managed efficiently. Raw images may have high pixel counts, and each pixel can be multidimensional and associated with additional data such as those derived from spectroscopy. In time-resolved studies, hundreds of tomographic datasets can be collected in sequence, yielding terabytes of data. Users of tomographic beamlines are drawn from various scientific disciplines, and many are keen to use tomographic reconstruction software that does not require a deep understanding of reconstruction principles. We have developed *Savu*, a reconstruction pipeline that enables users to rapidly reconstruct data to consistently create high-quality results. Savu is designed to work in an ‘orthogonal’ fashion, meaning that data can be converted between projection and sinogram space throughout the processing workflow as required. The Savu pipeline is modular and allows processing strategies to be optimized for users' purposes. In addition to the reconstruction algorithms themselves, it can include modules for identification of experimental problems, artefact correction, general image processing and data quality assessment. Savu is open source, open licensed and ‘facility-independent’: it can run on standard cluster infrastructure at any institution.

## 1. Motivation

### (a) Meeting a need from the user community

The use of X-ray tomographic imaging has increased dramatically in recent years, with researchers from a broad range of scientific disciplines gaining insights from its use. Repeated tomographic data collections (time-resolved studies) or tomograms with additional information at each voxel (such as crystallographic or chemical information) are also now possible at synchrotron sources. The raw data collected in a single experiment can reach several terabytes, which then require reconstruction and analysis. There is a need to reconstruct both rapidly and at high quality. Ideally, reconstructions can be generated quickly enough that they can be carried out during experiments and thus be used to guide how the experiments are performed. Analysing tomograms (segmentation, measurements, modelling, etc.) can take months or years; improvements to the quality of reconstructions can, therefore, bring great advantage not only to the quality, but also to the efficiency of scientific output.

Many researchers performing tomography do not have—and should not need—specialist knowledge of reconstruction methodologies in order to obtain good results and also benefit from recent advances in data-processing techniques. Reconstruction software should, therefore, be flexible enough to be customizable by an experienced user, while being intelligent enough to apply suitable processing steps with minimum user input. A modular solution can address these competing needs while also allowing novel algorithms to be incorporated as they are developed. A solution which is open source and facility-independent while maintaining cross platform capabilities would be beneficial, as a wider user base encourages development and collaboration.

The tomography community at Diamond Light Source (DLS) is steadily growing, with more beamlines coming online which are either dedicated to tomography or make use of it as one of a suite of techniques (beamlines I12, I13 and I18 are operational, and B24 is under construction). The community is collaborating to produce common tools that can be used on all beamlines, providing users with as productive an experience as possible.

### (b) Success of computational streamlining with other synchrotron techniques

The data deluge seen at modern tomography beamlines is also being experienced with other techniques, including macromolecular crystallography (MX). In MX, there has been significant effort spent on automation of both data collection and processing [1]. At present, routine experiments can be conducted with minimal user input, and more complex experiments are made significantly more straightforward by the array of available software tools. There is no substitute for a clear understanding of experiments, but such automation allows experienced users to process and evaluate data at substantially increased speeds. The advances made on MX beamlines at DLS have had a clear impact on the scientific output of this community, and it follows that to deal with the large quantities of data which are now being routinely collected on tomography beamlines, automation is key.

## 2. Data collection and processing challenges

The above-specified motivations present many complex challenges for the scientists who run and develop a modern synchrotron tomography user facility. Overcoming the challenges in theory requires optimal solving of scientific problems (e.g. understanding causes of and finding means of effectively dealing with artefacts). Overcoming the challenges, in practice, requires good software development. This section details the scientific challenges and their theoretical solutions, and §3 covers the practical implementations.

### (a) Tomographic techniques with demanding data-processing requirements

The basic tomography experiment requires obtaining some information along each of a large number of rays through a sample, from different angles. Reconstruction, or finding the properties at each spatial point in the sample consistent with the observed data, is handled by computer algorithms. Equally spaced data measuring a single property at equal angles along parallel rays is a simple case computationally, but the type of information, experimental constraints, artefacts or sheer volume of data can result in even this being a complicated task for the computer systems.

#### (i) High pixel array imaging

Digital cameras with large pixel arrays can produce datasets so large that state-of-the-art computational resources are required to manage them. For example, at DLS beamlines I12 and I13, the pco.4000 (PCO AG, Germany) imaging detector is used for tomography. It can produce 16-bit images of 4008×2672 pixels at 5 Hz, and therefore, yield a 160 GB dataset in about 45 min.

#### (ii) Phase contrast tomography

Phase contrast by propagation occurs naturally with the coherent X-rays produced by a synchrotron. Using a single image at each angle, back-propagating the wave function [2] allows one to remove the effects of phase contrast without much computational expense. Quantitative information about the phase shift introduced by different materials in the sample may be retrieved by using multiple images as in Vo *et al*.'s method [3], requiring many iterations to process the data.

#### (iii) High-speed, time-resolved tomography

The newer ‘scientific’ complementary metal-oxide-semiconductor detectors such as those used in the pco.edge 5.5 detector (PCO AG, Germany) can record at 100 Hz. With 11 MB full-frame images, the camera can record data continuously at over 1 GB s^−1^. Very high speed detectors (e.g. the pco.dimax (PCO AG, Germany) and Phantom Miro (Vision Research, USA)) can record full-frame images at over 1000 Hz. While they must normally acquire in batch mode and download in between batches, the Swiss Light Source has developed a continuous recording capability for their high-speed detector. Novel algorithms, such as that described elsewhere in this journal issue [4], can make use of temporal information to improve the spatial resolution, but require many iterative steps on corresponding slices drawn from several successive volume reconstructions.

#### (iv) Chemical tomography

There is a growing need for spatially resolved chemical information. Although X-ray absorption tomography can reveal a great deal of information about a sample, the amount of chemical information it reveals is limited. While attenuation of the X-ray beam through a sample varies according to the atomic number of the element(s) in its path, this alone cannot be used to discriminate between elements of similar atomic mass or give definitive indications of chemical composition. X-ray fluorescence (XRF) [5,6], X-ray diffraction (XRD) [7] or X-ray absorption near edge spectroscopy (XANES) [6] data can be obtained so as to be suitable for tomographic reconstruction—yielding chemical, structural and electronic information at each point in the sample.

It can be advantageous to bin raw chemical tomography data (average neighbouring pixels), so that reconstruction can be performed on a standard desktop computer during an experiment. This enables tomograms to be used to guide experimental strategy. Reconstruction without binning is much more time consuming, and benefits from a computational cluster.

### (b) The NeXus format

Data at DLS are saved in the standard NeXus format [8]. This allows information about the collection strategy, type of data, set-up geometry and the results of each analysis step to be recorded (even for various types of bespoke experiment) and used for the reconstruction process. This information can be used for a great variety of experimental set-ups, including the challenging scenarios described above. There are also technical advantages to NeXus which are detailed later in the article.

### (c) A modular workflow enables processing strategies to be varied

The manner in which data are optimally processed depends upon the data themselves and what the reconstructions will be used for. The techniques and parameters which best remove artefacts and enhance features for one dataset may be different to those that produce the best-quality results with another. While some tomograms are used for analyses that will take months, others are used for humbler tasks such as visual examination or checking the effectiveness of an experimental set-up.

The data-processing pipeline is therefore modular. If users wish to move away from a default workflow, they will be able to choose which processing features to use and how to use them. There is of course a tension between the desire to generate reconstructions of high quality and the wish to make reconstruction a rapid and easy process; the ultimate aim of Savu is to enable both.

### (d) Dividing the problem

**Figure 1.**
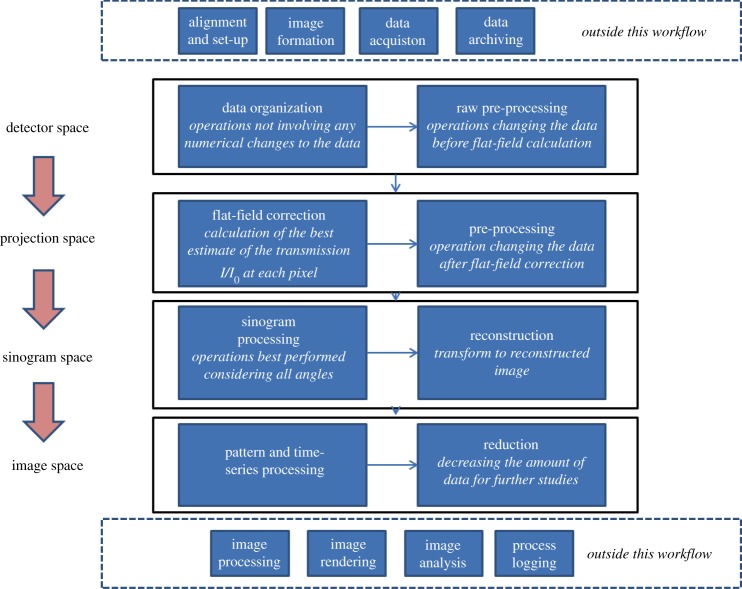
Schematic of the data-processing pipeline, in context of other steps required for a complete experiment.

Many of the processing steps may be applied to a small subset of data, enabling computational parallelization. However, the different steps require different sets of information; each problem has a specific *geometrical space* (detector, projection, sinogram or image) of applicability. The geometrical space that the data are represented in is a useful categorization, as it relates directly to the organization of the data in memory and hence to the efficiency of accessing those data for computation. The conceptual division of the problem for the data pipeline design is illustrated in figure 1.

To obtain the best final results, it is important to apply artefact corrections in the right order; if this is done in the wrong order, avoidable degradation of data quality can occur. For computational efficiency, it is sensible to minimize transfer between geometrical spaces.

#### (i) Detector space

Operations in this domain are intended to obtain the best estimate of X-ray flux reaching each pixel of the detector. Operations that involve changing the value of the data at each pixel, or rejecting pixels entirely, are performed. This includes correction for faulty elements in the detector (i.e. ‘dead’ or ‘hot’ pixels whose value does not represent the photon flux reaching that element) and X-ray strikes producing artificially high brightness values. Areas of the image known to contain spurious items (such as parts of the experimental equipment that do not rotate) can be removed or masked. Pixels relating to data collected from multiple samples may be summed or averaged. Operations in detector space should avoid spatial interpolation of pixel values as this will spread noise.

#### (ii) Projection space

Moving to the projection space domain requires transforming the coordinates of the raw data to a regular grid in the coordinates of the laboratory. Various processes can be performed to obtain the best estimate of X-ray attenuation as relevant to each regularly spaced ray in laboratory coordinates. The detector optics at Beamline I12, for example, have significant optical distortion, and further processing steps are simplified if the distortion is corrected at this stage. Regions on the scintillator that are defective or obscured by dust or scratches can be masked or replaced. Depending on a beamline's capabilities, intensity may be normalized via electronic supplementary material (e.g. ion chamber readings), with these readings automatically obtained from the NeXus data file. Applying phase-propagation-based processing such as those described by Paganin *et al.* [2] or Vo *et al.* [3] requires two-dimensional Fourier transforms of real space images, and such transforms can also be performed at this stage.

#### (iii) Sinogram space

Transforming from projection to sinogram space does not involve calculation of new values, but involves extracting data from every projection to make each sinogram. Here, we organize the data so as to obtain attenuation as a function of sample rotation angle. Computationally, one must take care to avoid this step being extremely slow. Calculations which benefit from or which require sinogram space are ring suppression by Fourier [9] or analytical [10] methods, refinement of the rotation axis position and tilt [11], tomographic reconstruction by filtered back projection or iterative methods, and the recently developed multiple time-frame reconstruction [4].

Calculation of rotation centres in sinogram space can be automated via a Fourier analysis method developed at DLS [11]. We have found that this method is robust in the presence of noise or low contrast features, allowing subpixel refinement of the alignment centre. Sinogram space is the best domain in which to address ring artefacts which result from errors in imaging systems (including dust and scratches on scintillators and optics). While ring artefacts can be partially prevented by correcting projection images with flat- and dark-field images, some removal is normally necessary. Because the feature in the images arising from a static defect is a perfectly straight line in the sinogram, frequency space methods [9] and analytical methods [10] can be used to reduce these artefacts.

#### (iv) Image space

By tomographic reconstruction, we find a value of the required property at each point within the sample, the results of which are classified as being in image space. For some experiments, rescaling the numerical value to reduce the bit depth for delivery to the researcher is appropriate, although at DLS the original data are archived and can be reprocessed if necessary. For example, time-series regularization (e.g. the method described by Kasantsev *et al*. [4] elsewhere in this journal issue) and the processing of reconstructed spectral or diffraction patterns (see below) is performed in this geometrical space. For the time being, we consider the subsequent steps of image analysis, segmentation, shape finding and classification to be outside the scope of Savu's workflow.

### (e) Using software to identify experimental problems

High-quality reconstructions rely upon high-quality data, which means that experimental problems (which can be numerous and varied in bespoke experiments with custom sample environments) must be identified. Software can help to identify these problems; experiments may then be modified accordingly, and where this is not possible experimental errors can sometimes be corrected for computationally. We use a simple means of identifying experimental problems, as follows.

The parallel beam of a synchrotron enables a complete dataset to be collected over 180°. We horizontally flip the last projection image and then align it to the first via template matching. In an ideal experiment, the resulting images would be identical. Visual comparison of the pair of images can rapidly reveal a surprising range of experimental problems; some of these are sufficiently subtle that they might otherwise go undetected. With Savu's modular system, the automated detection of problems could also be added in the future. Experimental problems identified by this method include the following.

#### (i) Sample deformation

Sample deformation is a common problem with a variety of causes. Non-rigid samples (often biological) may relax by themselves over time. The high flux of a synchrotron X-ray beam can cause samples of sufficiently low melting temperature to deform via heating (electronic supplementary material, figure S1). Outright beam-induced damage is also possible, particularly with biological samples. Bubbles may form owing to X-ray interactions with the sample, and these may expand and move, pushing other material aside. Rigs which apply compressive, tensile and torsional forces can deform samples (electronic supplementary material, figure S2). Some deformation may be unavoidable, but identification of such problems allows one to try to minimize them. Methods for minimizing deformation artefacts include: reducing overall flux; filtering the beam to reduce the flux of the strongly interacting lower-energy photons; introducing relaxation periods prior to scanning of non-rigid samples; and decreasing scan times used with compressive rigs. Optimal imaging of non-rigid biological samples (e.g. joints) in compressive rigs is particularly tricky as it requires a balance to be struck between competing needs: the reduction of deformation artefacts (achieved with long relaxation periods and short scan times); the minimization of beam damage (attained with short scan times with filtered X-rays of overall low flux); the maintenance of good signal:noise (achieved with long scan times and high flux) and good contrast (which for some beams benefits from minimal filtration); and the number of scans one can perform in a beamtime (which benefits from minimal relaxation periods and short scans). Investigating how best to balance such competing needs would form the basis of a useful methodological study.

#### (ii) Movement of samples

If alignment of projections collected 180° apart requires vertical translation, one can deduce that vertical sample movement has likely occurred. With good-quality stages, movements sufficient to affect microtomography results are rare, although they can be caused by cable drag. However, in nanotomography (which uses X-ray optics to increase resolutions to tens of nanometres) stage instability is a significant problem. Rotation stages are rarely stable on the scale of tens of nanometres. Non-synchrotron nanotomography machines commonly address this problem either by translating images according to physical measurements of stage movements, or by making translations based upon comparison with a repeat scan. In the latter scenario, after a dataset has been recorded the sample is again rotated and images are collected every few degrees; corrections for stage movements are calculated on the basis of alignment and interpolation. In electron microscopy cryo-tomography, the tracking of gold particles is a common solution. At DLS, we have had some success with particleless tracking; each image is aligned to its predecessor by matching of inherent features. Any of these solutions could be included in the new processing pipeline.

**Figure 2.**
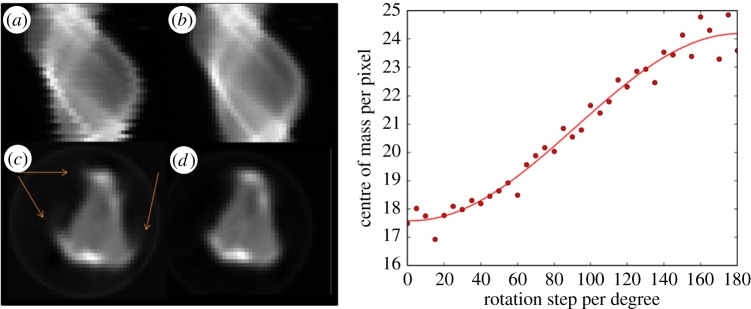
(*a*) Raw sinogram data demonstrating effect of motor hysteresis. (*b*) Aligned and centred sinogram. (*c*) Reconstruction of raw sinogram with artefacts highlighted. (*d*) Final reconstruction of corrected sinogram. Right: Determination of centre of mass, required shift per row and centre of rotation adjustment for sinogram correction. Adapted from Price *et al.* [6] with permission from the PCCP Owner Societies.

With mapping scans such as those used in chemical tomography, translational motors can experience micrometre or submicrometre hysteresis. For an experiment with a resolution of tens of micrometres, this is not significant, but when conducting microfocus tomography experiments such as those on DLS Beamline I18, where the translation step can be as small as 1 μm, this hysteresis can have significant effects on the quality of raw data. The sinogram in figure 2 is notably misaligned from one row to the next. Some simple image processing (centre of mass corrections) using Python v. 2 libraries can correct for this before the reconstruction stage. The effect of such hysteresis is seen in figure 2*c*. In this instance, by aligning the fitted centre of rotation (by fitting a sine function through the centre of mass of each row) with the centre of the sinogram, simple horizontal translations are all that are required for correction.

#### (iii) Tilts

Samples should rotate around an axis parallel to the vertical axis of the detector. If this condition is not satisfied, the rotation axis is said to suffer from either out-of-plane or in-plane tilt. The latter leads to a rotation centre which varies across the image, and a surprisingly small error can affect results significantly. For example, an in-plane tilt of just 0.05° produces a difference of two pixels in the rotation centre between the top and bottom of a full-frame image from the pco.edge 5.5 detector; two pixels is an error sufficient to degrade reconstruction quality to an obvious extent. Both in-plane and out-of-plane tilts can be identified via comparison of images collected 180° apart (electronic supplementary material, figure S3).

### (f) Chemical tomography

There are various challenges associated with chemical tomography, and some of these are shared with the collection of an additional dimension of data (e.g. time or temperature) in non-chemical tomography.

Data collection challenges in chemical tomography include problems such as the penetration depth of X-rays (notably for XRF–computed tomography (CT) and XANES–CT), self-absorption of fluorescent X-rays, and the time required to collect a spectrum or pattern with sufficient signal:noise. Attempting to collect chemical tomography data *in situ*, or at operating conditions, increases all of these challenges. For example, there is an increase in both scatter and absorption from the additional reactants and reaction cells.

The penetration depth of X-rays is dependent on the material they are travelling through. For absorption and diffraction CT conducted with high energy X-rays (more than 50 keV), attenuation length is sufficiently large that samples can be in the 1–100 mm range. However, when measuring XRF–CT (or indeed XANES–CT), measurements are limited not by the penetration depth of the incident X-ray beam but rather by that of the weaker-penetrating, lower-energy fluorescent X-rays. For example, while a 50 keV beam would penetrate 400–500 μm of pure iron, the 6400 eV K_*α*_ fluorescence of the iron would only be able to escape approximately 8 μm. Conveniently, many samples of interest for XRF–CT, however, contain small amounts of high atomic number (Z) elements in a low Z matrix, and therefore, the practical sample size can be larger, e.g. 100–1000 μm [5,6].

Self-absorption is particularly significant in XRF–CT owing to the short attenuation lengths of the fluorescent X-rays. Concurrent collection of absorption data, for example with a transmission ion chamber or imaging camera, allows for an estimation of sample absorption, although Rayleigh and Compton scatter from the fluorescence may also be used [12,13]. Because this information is included in the same dataset as the tomography data, the benefit of using a pipeline such as Savu is that the corrections can be included as a simple module option.

The time taken to collect a projection in full field XANES–CT can be fast (tens of milliseconds per projection) and comparable to that of absorption CT. The collection of data for mapping XRF–CT and XRD–CT, however, is slower (tens to hundreds of milliseconds per point), and a single projection can take many minutes to collect. This is due to the time required to collect a sufficient number of counts and wait for sample stage movements and detector readouts associated with each point of a map. Improvements in detectors, such as the recent Maia system, mean that fluorescence data can be collected an order of magnitude faster, increasing the prospect of scanning XRF–CT and XANES–CT becoming routine techniques [14].

## 3. Proposed processing pipeline solution

The practical implementation of the steps outlined above is illustrated in table 1. It outlines the nature of the steps in a tomography processing scenario and which geometrical space the calculations take place in.

**Table 1.** Workflow of the standard DLS pipeline usage, showing specific steps required for standard absorption tomography and alternate steps that would be required for other modes.

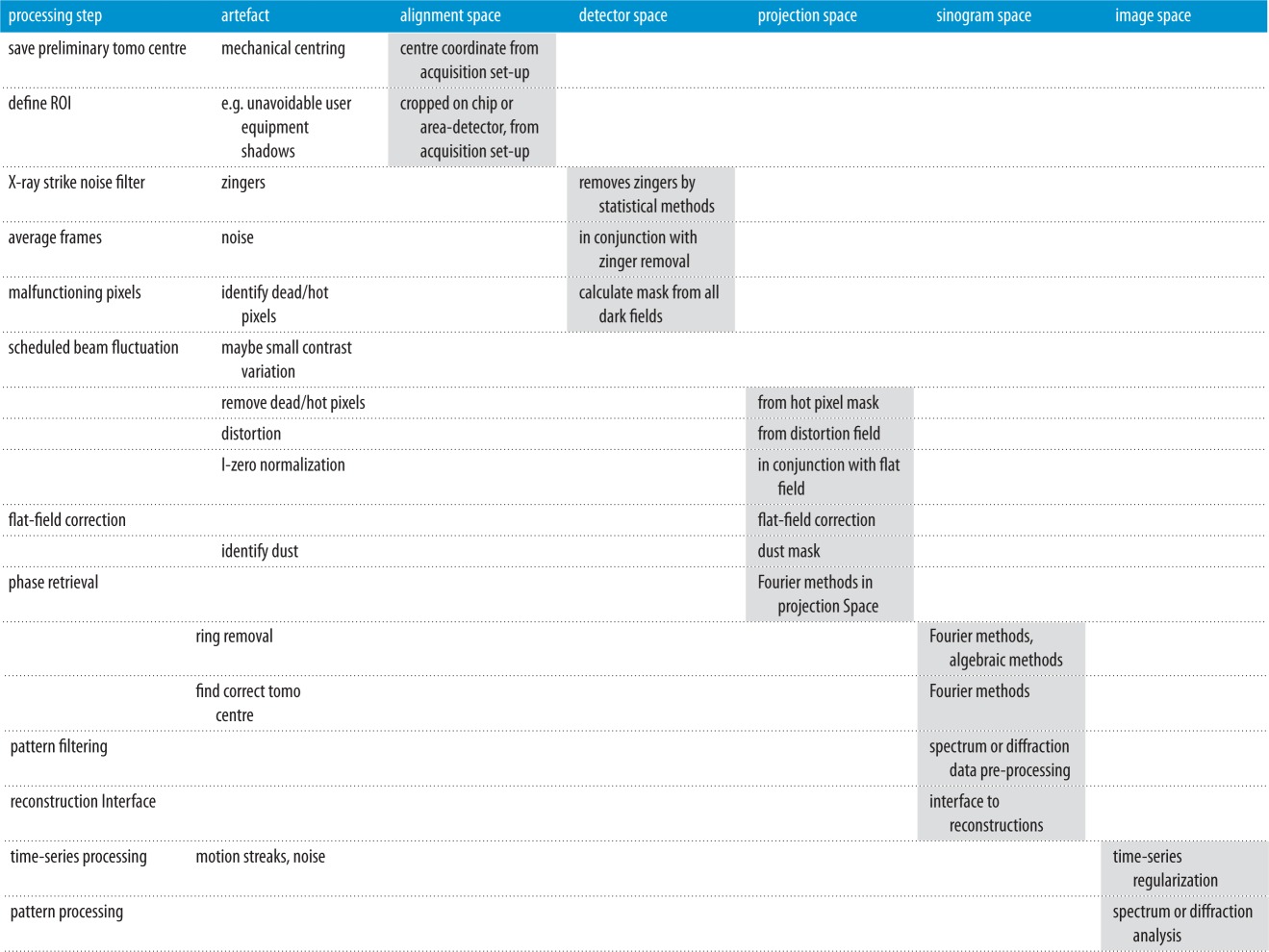

### (a) Technical solutions

We have presented a variety of challenges which modern synchrotron beamlines have to manage in order to make the most efficient and effective use of available beamtimes. At DLS, all the tomography beamlines work together with the aid of the Data Acquisition and Data Analysis Groups to provide practical solutions to these many difficulties. This work is always in flux as detectors and processing methods are constantly being updated, so the solutions presented here are a snapshot of items currently being developed. This article is focused on software solutions to data management and processing; how data are harvested from the detectors is covered elsewhere in detail [15]. Because a specialized IT infrastructure [16] is required to manage the vast quantities of data collected and processed at DLS, many of the software solutions presented here are tailored to make optimal use of this infrastructure.

#### (i) NeXus (NXtomo)

**Figure 3.**
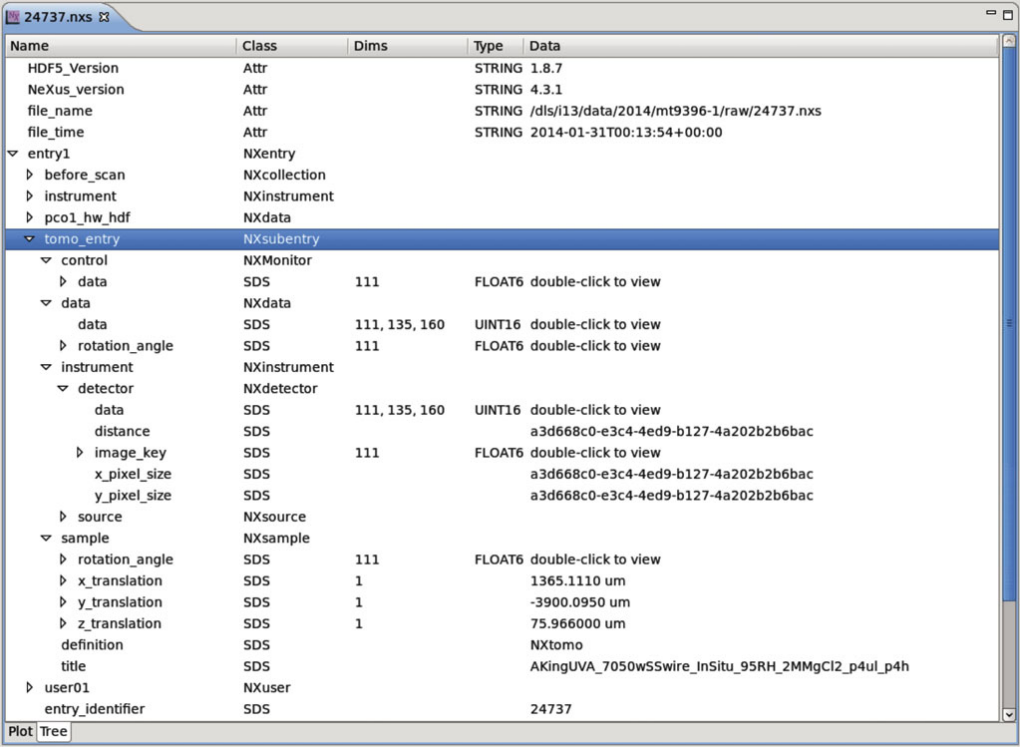
NeXus file structure showing the NXtomo subentry expanded to show information such as motor positions, detector data and settings, and scan title.

The NeXus format [8] has been selected by DLS as the primary means of storing data. The standard is mature and becoming more accepted by the tomography community. Together with the HDF5 backend [17], NeXus brings performance benefits for the parallel file systems at DLS. These systems are optimized to deal best with a small number of large files, as opposed to a large number of small files, and the multidimensional nature of NeXus and HDF5 plays to these strengths. The choice of a standard format for all data collections is critical for developing the cross-beamline logging, archiving, reconstruction and processing solutions presented here. Specific to tomography is the NXtomo entry type [18], which fully specifies the data required to reconstruct a final volume, as shown in figure 3.

#### (ii) ISPyB

As the number of data collections in a typical visit to a beamline increases (a recent time-resolved study on DLS Beamline I13 involved 3000 tomographic scans being performed in 5 days), it becomes increasingly difficult to satisfactorily record information about the scans manually in a log book. This is a problem which has already been addressed by the MX community [19]; because DLS uses common support groups, it has been relatively easy to transfer the logging techniques used in MX to tomography. Although not in routine use by users, the concept has been prototyped and is running for all tomography data collections at DLS. Whenever a tomography scan is conducted, the NeXus file is used to populate the ISPyB database with information about the scan, such as the rotation angles, the user-defined title for the scan, projection frame sizes and unique scan identifiers. These items are inserted into the standard MX tables, but proposals already exist for further development of ISPyB to allow more specific data to be held, such as information on post-processing.

**Figure 4.**
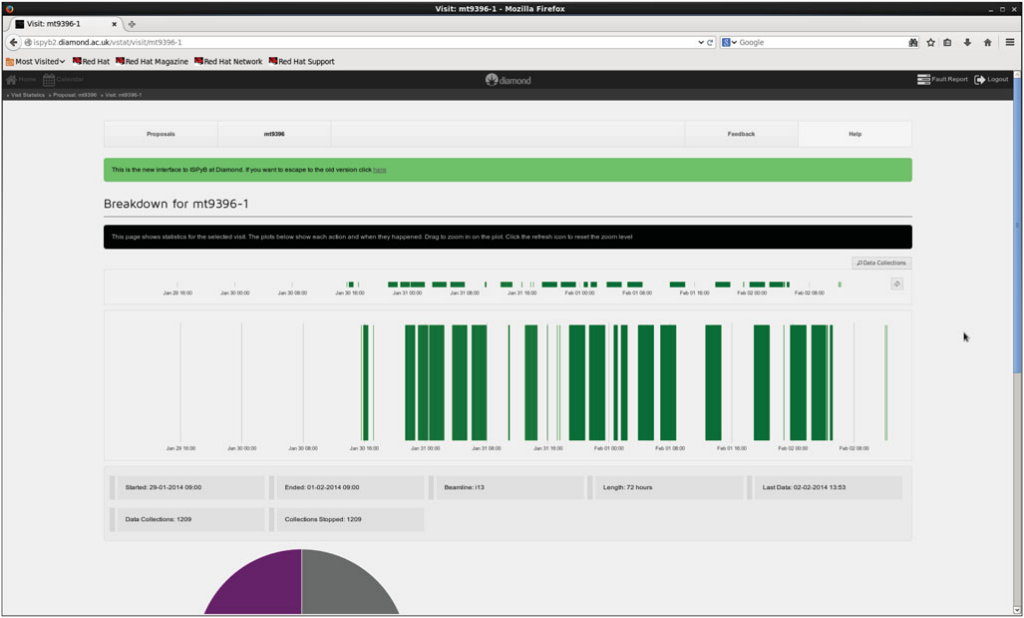
Synchweb tomography visit overview showing when data collections took place during the beamtime.

**Figure 5.**
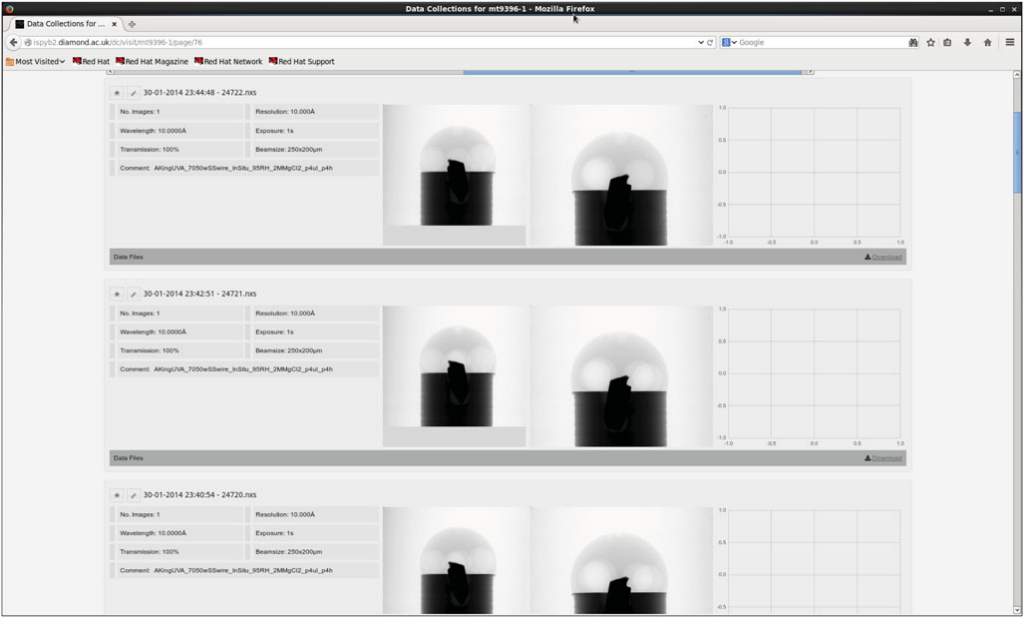
Detailed Synchweb webpage showing specific scan information, as well as corrected projections taken from the data files themselves. The empty section on the right of each data collection is a placeholder for a reconstructed slice once it has been generated.

Once the database has been populated, it can be viewed from a web browser (figures 4 and 5). This allows researchers to see which data have been collected and obtain some indication of their quality.

#### (iii) Long-term data preservation

**Figure 6.**
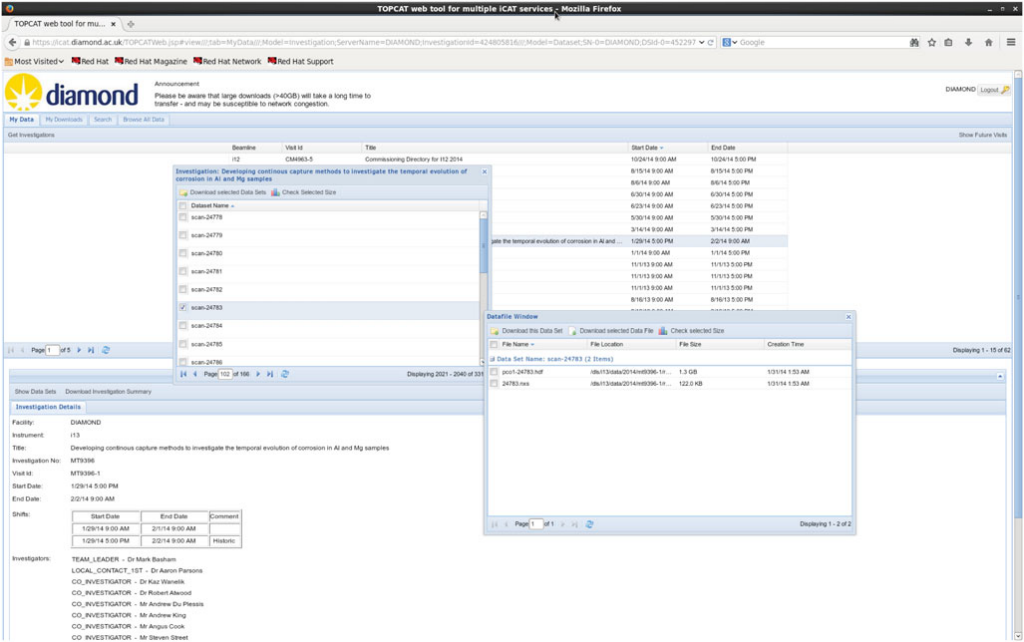
Example of the TopCat ICAT web interface for downloading datasets after they have been archived to tape.

Long-term storage of raw and processed data is critical. Many funding bodies now call for data to be archived reliably, and it can be useful for readers and reviewers to have access to raw data and the information used to generate published results. For tomography experiments in which data collected for a single tomogram can be 100 GB in size, it is not always easy for individuals or universities to host such data long-term. At DLS, we use a data archiving system which makes use of the ICAT [20] data management system. All raw data and some processed data are archived, and are accessible from a web browser (figure 6) via which both individual data items and groups of items can be downloaded. Data are accessible only by the researchers who collected them, unless they choose for them to be openly available.

### (b) Reconstruction pipeline: computational challenges

The above-described reconstruction pipeline presents many difficulties for a computing system. Translating large tomographic datasets (routinely around 100 GB per scan) orthogonally and applying various modular processing steps requires careful data management. At DLS, we generally use about 20 parallel computer nodes (each with two Tesla graphics processing cards (Nvidia Corporation, USA)) to process data. With tomo-recon (see below), it currently takes about 20 min to reconstruct a standard tomographic dataset.

The processing of tomographic datasets from a parallel beam source is well suited to cluster computing. Projections and sinograms can be processed one at a time (or in small consecutive bunches) in an arbitrary order; for example, each sinogram can be reconstructed independently to form a tomographic slice. In even a simple pipeline, such as tomo-recon, this can allow processing to be split, giving each computer node only the portion of the dataset it requires. However, when processing data in an orthogonal fashion (switching between projection and sinogram space), appropriate data need to be transmitted between nodes as the pipeline processing progresses; this adds complexity to the problem. Furthermore, there are some processes which require specific regions of data to be read non-sequentially from the data block (e.g. blob removal which requires frames 180° apart); these regions are spread through the file and therefore slow to access.

Developers of scientific modules should be unaware of the underlying complexities of the pipeline, so that their programming is kept as easy as possible.

### (c) Reconstruction pipeline: implementations

#### (i) Current approach: tomo-recon

**Figure 7.**
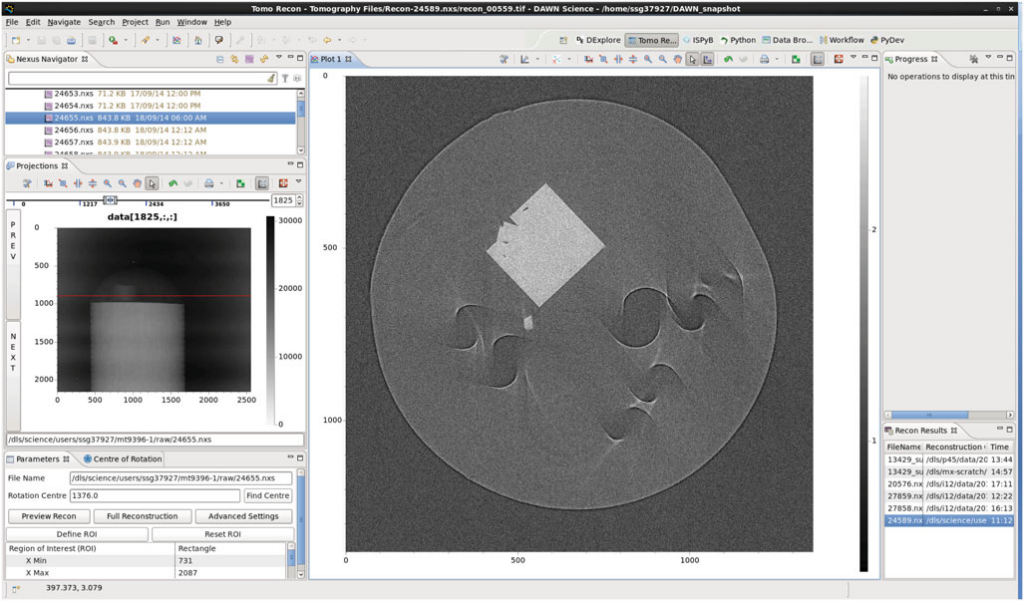
DAWN's tomography reconstruction GUI being used with the tomo-recon backend. It shows single reconstructed slices which provide feedback during experiments and help the user to choose parameters for the full volume reconstruction.

Currently, most reconstructions at DLS are reconstructed, at least initially, using the tomo-recon pipeline. This is mainly conducted via a graphical user interface (GUI) which is integrated in the Dawn [21] visualization and imaging software used across beamlines at DLS (figure 7). tomo-recon is a set of Python v. 2 scripts [22,23] which uses the Diamond Clusters Grid engine to batch process in parallel. This makes use of the ability to slice HDF5 datasets in any direction, as the batch processing is done entirely on sinograms. This brings some great performance improvements, although the projection space methodologies described above are not possible with this method. Another drawback of tomo-recon is that it can only output reconstructions in TIFF format. Writing a NeXus file for the output would provide a much cleaner (only one file) and richer (added metadata) solution.

#### (ii) New approach: Savu

Savu is a Python v. 2 project which makes significant use of existing stable libraries to solve the complex problems described above with a minimal and clear implementation. It is opensource and freely available on Github [24]. It makes use of the Python package index PyPI, so that it is trivial to install in Python using ‘pip install savu’.

Savu makes use of a plugin-style architecture, whereby each plugin (or module) represents a step in the workflow shown in table 1. This allows for a clear division between the framework (which deals with cluster and data management issues) and the scientifically relevant plugins. Beamline staff and users can customize the processing pipeline extensively by choosing which modules and associated parameters to use. Citations relevant to each plugin are included in the output to enable easy referencing.

Data produced by a plugin are presented to the next plugin via an array-like interface. This enables the next plugin to choose which data it uses. Data may need to be accessed from any cluster node, and this is handled by the PHDF5 extensions in h5py using mpi4py. The H5 element in these extensions is convenient for our purposes, as we input and output data in the HDF5 format. If we find that this simple system for transferring data is not performant enough, we will replace it with, for example, the message passing interface (MPI), ZeroMQ or Database interaction. Savu is therefore resilient to inevitable changes in both software and hardware; the underlying engine may have to change, but the core interface to the data should not.

Data output from plugins are saved as intermediate HDF5 files of three specific types (RawTimeseriesData, ProjectionData and VolumeData), each of which is based on the requirements of the pipeline. RawTimeseriesData is equivalent to detector space; ProjectionData is equivalent to both projection space and sinogram space, as in HDF5 they are effectively the same; VolumeData corresponds to image space. Each plugin takes one or more of these types as input, and returns one as output. For example, a reconstruction plugin will take ProjectionData as input, and return VolumeData as output. Most tomography pipelines will go ultimately from RawTimeseriesData to VolumeData, whereas radiography experiments will stop at ProjecionData. To save disk space, intermediate HDF files will normally be deleted when no longer needed by the pipeline, but they may optionally be kept so they can act as a log and be checked for errors and anomalies.

**Figure 8.**
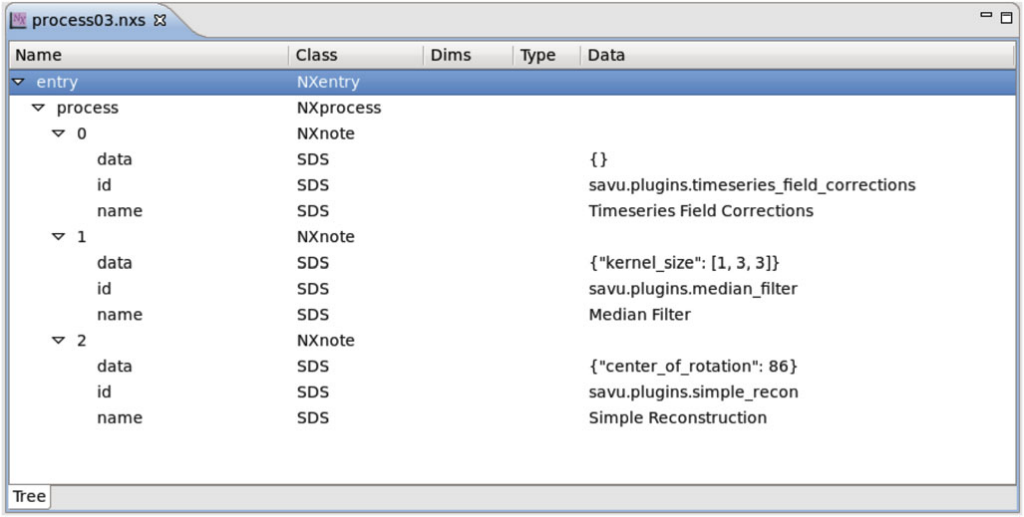
Process configuration as stored in HDF5 file displayed in DAWN.

The Savu framework incorporates runners that use the plugins and data to run the processing pipeline. The processing to be carried out is recorded in an HDF5 configuration file (figure 8). This contains the list of plugins to be executed in order, along with any user-defined parameters. The reason for choosing HDF5 for this format, which is not so convenient to edit, is that the full processing configuration can be easily stored within every processed file, preserving the processing provenance. Although not currently necessary at DLS, if tomograms need to be deleted to free up disk space, they can be easily recreated if needed.

MPI and mpi4py were chosen for running parallel jobs, as MPI is available on most cluster and supercomputer systems, and it scales well to running processing on very large systems. It is also a tried-and-tested system with many years of development by an active user community.

Data processing is performed by plugins, which are written in Python without the need for additional libraries. Each plugin has access to data from the previous plugin, and to the directory in which it will save results. Plugins follow a hierarchy, which allows new plugins to make use of more complex plugins (e.g. filters and reconstruction modules) which simplifies code writing; this provides the author of a simple plugin with a high-level interface. A code snippet showing the implementation of a three-dimensional median filter is available from the Savu Github site [24].

In summary, Savu is a lightweight Python framework which splits the problem of dealing with complex data processing into two. Core processing and hardware management are kept separate from the scientifically relevant plugins. This allows software specialists to update the framework as required without needing to update modules. The plugins themselves can therefore be created and improved without concern for the underlying infrastructure.

## 4. Conclusion

DLS currently processes large tomographic datasets of different types. Data collection and processing, experimental logging and data archiving are computationally efficient. DLS provides a GUI for reconstructions which is commonly used for simple X-ray absorption reconstructions. However, there are several points at which user intervention is required, and storage in traditional image formats such as TIFF does not efficiently make use of modern high-volume storage systems. Even adding relatively simple new steps requires unnecessarily large temporary storage and unwieldy numbers of files to manage.

In order to rectify this, a standard data and metadata format (NeXus) based upon HDF5 has been adopted at DLS, and is now in use across all tomography beamlines. The existing reconstruction pipeline (tomo-recon) is currently being replaced by Savu. This Python 2-based pipeline can output reconstructed files in the NeXus format, and it is modular: users can control the processing workflow, and new modules can be easily incorporated. Savu is facility-independent, and in the process of being adopted by other facilities such as the pulsed neutron and muon source ISIS (http://www.isis.stfc.ac.uk/). It is being distributed by the Collaborative Computational Project in Tomographic Imaging (CCPi; http://www.ccpi.ac.uk/).

## Supplementary Material

Electronic Supplementary Material-Figure S1

## Supplementary Material

Electronic Supplementary Material-Figure S2

## Supplementary Material

Electronic Supplementary Material-Figure S3
