# Supplementary figures and images for: A high-throughput system for high-quality tomographic reconstruction of large datasets at Diamond Light Source
Source: Philos Trans A Math Phys Eng Sci. 2015 Jun 13;373(2043):20140398. doi: 10.1098/rsta.2014.0398 (PMC4424489; doi:10.1098/rsta.2014.0398)

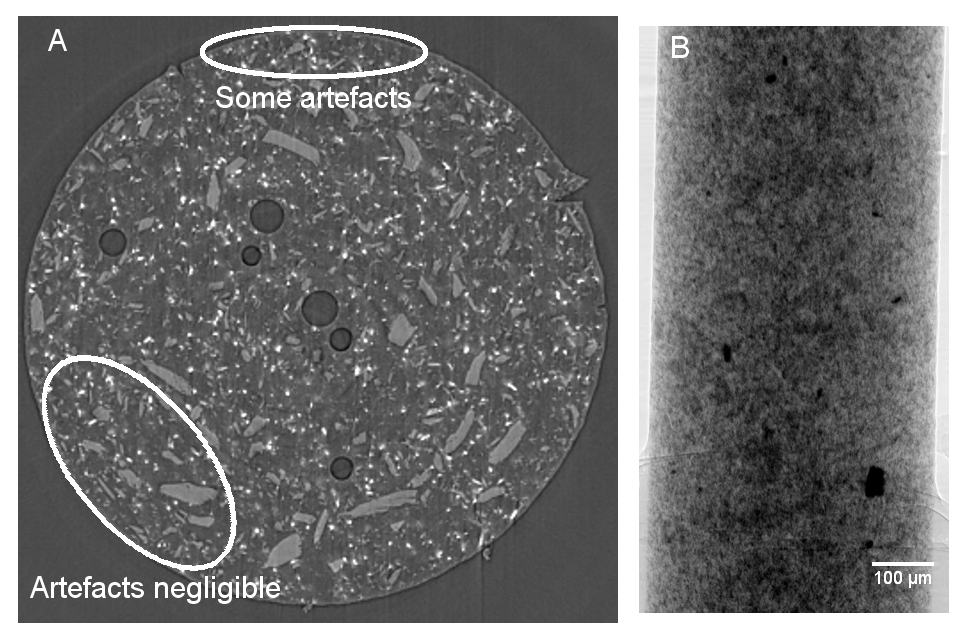

Supplement: Electronic Supplementary Material-Figure S1 [file rsta20140398supp1.gif]

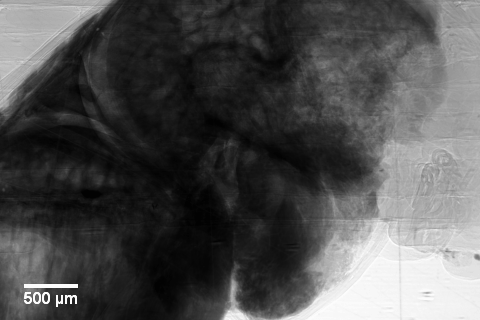

Supplement: Electronic Supplementary Material-Figure S2 [file rsta20140398supp2.gif]

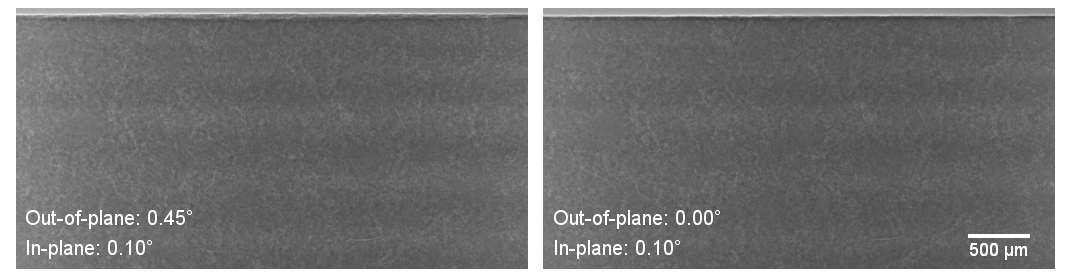

Supplement: Electronic Supplementary Material-Figure S3 [file rsta20140398supp3.gif]
